# Supplementary material for: Rapid Preparation of Novel Ionic Polymer–Metal Composite for Improving Humidity Sensing Effect
Source: Polymers (Basel). 2023 Jan 31;15(3):733. doi: 10.3390/polym15030733 (PMC9921886; doi:10.3390/polym15030733)
Supplement: Supplementary file 1 [file polymers-15-00733-s001.zip › polymers-2160108-supplementary.pdf]

Supporting information

Rapid Preparation of Novel Ionic Polymer–Metal Composite for Improving Humidity Sensing Effect

Chun Zhao †, Yujun Ji †, Gangqiang Tang , Xin Zhao , Dong Mei , Jie Ru , Denglin Zhu and Yanjie Wang *

Jiangsu Provincial Key Laboratory of Special Robot Technology, Hohai University, Changzhou Campus, Changzhou 213022, China

* Correspondence: yjwang@hhu.edu.cn

† These authors contributed equally to this work.

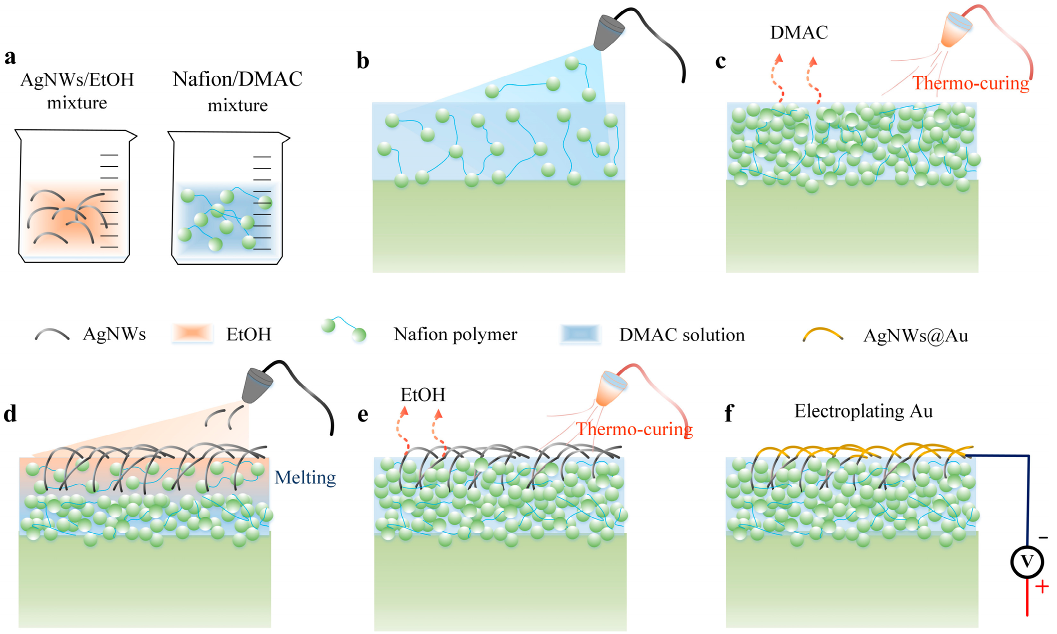

Figure S1. Preparation process of steps (c) and (d)

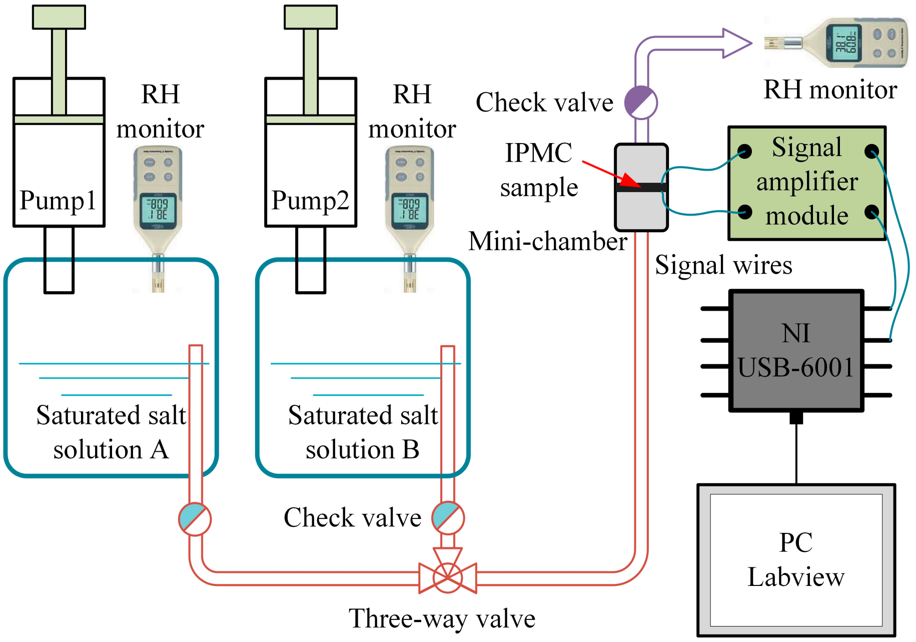

Figure S2. Humidity measurement setup

The ionic electrical measurement platform is shown in Figure S2. Saturated salt solution was filled into two humidity tanks A and B. Tank A was a stable humidity of 57 % RH, and different humidity gradients were achieved by changing the humidity gas in tank B. Tank A and tank B are connected to two ends of a three-way valve by pipes, the other end of which is connected to the mini-

chamber. In order to prevent backward flow, two check valves are set at the front and rear ends of the cavity. During measurement, air pump 1 was started first and the gas in tank A with relative humidity of 57 % RH was pumped into the chamber. It was ensured that IPMC contacts with gas A for several hours until its voltage response value is stable. Then the three-way valve was adjusted to pump the gas in tank B into the chamber. Due to the instantaneous switch of humidity, ionic electrical response will be detected between two sides of the sample. The entire voltage data was recorded on LABVIEW platform of PC via a data acquisition card of NI USB-6001. Due to the small magnitude of the generated electrical signals, a signal amplifier module was set up ahead of acquisition, and the generated electrical signals were amplified by 50 times. In order to ensure the reliability of the humidity in the chamber, three RH monitors were placed in the humidity tanks and the final exit to detect the humidity level of the gas into and out of the chamber in real time.

Table S1. Comparative table of different methodology

	Pd based IPMC	Ag NWs based IPMC	This work
Stability	High	Low	High
Surface resistance	~0.5 Ohm/sq	~8 Ohm/sq	~3.4 Ohm/sq
Sparse gap structure	No	Yes	Yes
Humidity sensing performance (57% RH - 100% RH)	0.41mV	9.6mV	7.3mV
